# Minimally guided bone regeneration procedure for immediate implant placement and provisionalization of a maxillary lateral incisor: A case report

**DOI:** 10.34172/japid.2020.002

**Published:** 2020-04-08

**Authors:** Abdulreza Fattahian, Farzaneh Poursafar, Siamak Yaghobee

**Affiliations:** ^1^Private Practice, Tehran, Iran; ^2^Department of Periodontics, Faculty of Dentistry, Tehran University of Medical Sciences, Tehran, Iran

**Keywords:** Bone regeneration, Dental implants, Dental restoration, Immediate dentalimplant loading, Single tooth, Temporary

## Abstract

Immediate implant placement has some advantages, such as time-saving, optimal soft tissue architecture preservation, and patient acceptance. In this case, prior to implant placement, minimally guided bone regeneration was performed to augment the concavity on the apico-labial aspect of a fractured maxillary right lateral incisor. After eight months, the tooth was extracted, and an implant was immediately inserted, and a provisional composite-based crown was delivered in the same appointment. After four months, well molded mid-facial gingiva and interproximal papilla were obtained, and a final metal-ceramic crown was fabricated.

## Introduction

Dental implants are a therapeutic approach for functional and aesthetic needs. Implant placement in the esthetic zone is one of the most significant challenges for clinicians.^1^ Different studies have demonstrated a high percentage of clinical success for immediate implant placement.^2^ The placement of an implant in the fresh extraction sockets reduces the overall treatment time and gives rise to satisfactory esthetic outcomes in most cases. However, a careful assessment of risk indicators of this procedure, such as thin tissue biotype, thin facial bone, and dehiscence of the facial bone is important.^3^ Immediate implantation provides the opportunity to deliver a provisional restoration. Numerous studies have reported good esthetic outcomes for such treatment.^4,5^

This case report presents the immediate implant placement and provisionalization in the anterior maxilla after a minimally guided bone regeneration procedure with a different flap design.

## Case report

**Figure 1:**
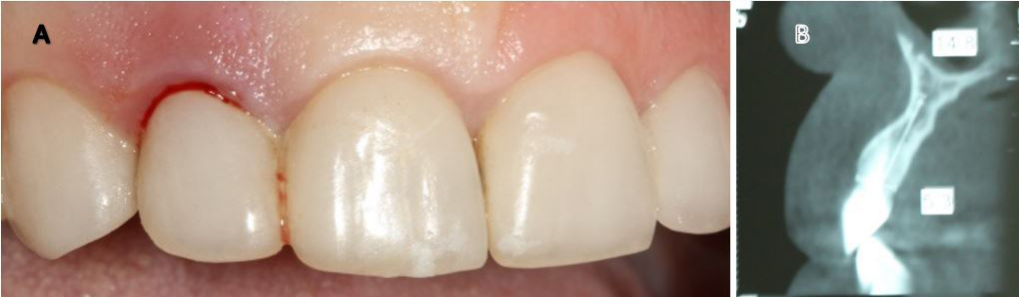
(A) The preoperative clinical and (B) CBCT views

**Figure 2:**
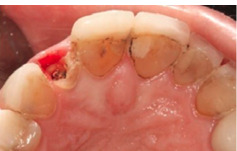
The fractured maxillary lateral incisor.

A 48-year-old female patient referred to the clinic interventions, immediate implantation with a complaint of the crown mobility and pain on biting related to the upper right lateral incisor (Figure 1). She was in good general health, and her medical history revealed no medical conditions. Intraoral examinations demonstrated healthy gingival and periodontal status, stable occlusion, acceptable intermaxillary relationship, and no parafunctional habits. Clinical intraoral examinations revealed an oblique complicated crown fracture of the maxillary right lateral incisor. The fracture line extended subgingivally on the labial aspect and ended close to crestal labial bone (Figure 2). Crown lengthening procedure was not a treatment option since it could lead to compromised esthetic outcomes. On the periapical radiograph, the tooth seemed to be an ideal candidate for immediate implant insertion; however, CBCT and clinical evaluations showed a large bone concavity in the apical area of the tooth (Figure 1). After a thorough clinical examination and radiographic evaluation, different treatment plans were explained to the patient. Taking into consideration the maintenance of an esthetic appearance during the treatment procedures and the patient’s desire for a minimal number of surgical the final treatment plan. The apical bone concavity had to be resolved; otherwise, implant placement inimmediate provisionalizatoin was decided upon as an ideal position was not possible; therefore, it was decided to augment the apical bone concavity before tooth extraction and immediate implantation. Prior to the surgical process, informed consent was obtained from the patient.

The treatment started with the placement of a prefabricated post and a composite core with a composite-based temporary crown (Dentocrown, Itena, Villepinte, France) on the lateral incisor.

### Procedure

#### stage 1

**Figure 3:**
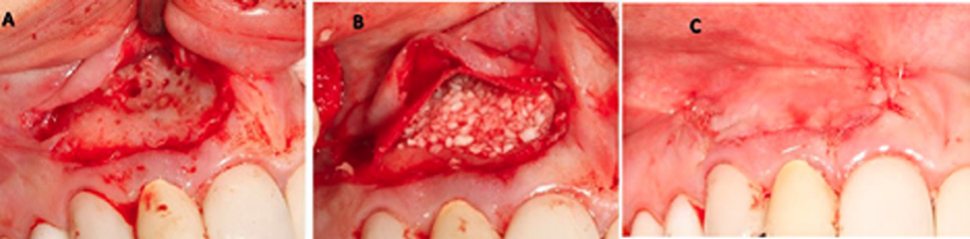
(A) The mucoperiosteal flap reflected to expose the apical bone concavity. (B and C) Guided bone regeneration procedure.

The patient was instructed to take 1 g of amoxicillin an hour before surgery. After administration of the local anesthetic agent (2% lidocaine with 1:100,000 epinephrine), the patient was instructed to rinse her oral cavity with 0.2% chlorhexidine gluconate (CHX) mouthwash for one minute. In this stage, the goal was to augment the concavity of the apical area without damaging the coronal hard and soft tissue architecture, especially the interdental papilla. A horizontal incision was made four mm apical to the free gingival margin of the maxillary right lateral incisor, continuing mesially to central incisor and distally to the canine to preserve the natural architecture of tissues around the tooth. Releasing incisions were made on the distal and mesial aspects. Then, a mucoperiosteal flap was elevated (Figure 3A). Cortical plate perforations were performed using a surgical handpiece to facilitate the migration of osteogenic cells and increase blood perfusion to the site. A particulate allograft (FDBA) (Cenobone, Tissue Regeneration Co., Kish, Iran) and a collagen membrane (Tissue Regeneration Co., Kish Tehran, Iran) were used to guide bone regeneration in this defect. The flap was closed primarily using 6-0 Glycolon sutures (Resorba Medical GmbH, Nürnberg, Germany) (Figures 3B and 3C).

After surgery, amoxicillin, 500 mg (q8h for seven days), gelofen, 400 mg (q6h for seven days), and 0.2% CHX mouthwash (twice daily for a week) were prescribed. The patient was asked to return at two-day, one-week, and one-month intervals after surgery for postoperative assessment.

#### stage 2

**Figure 4:**
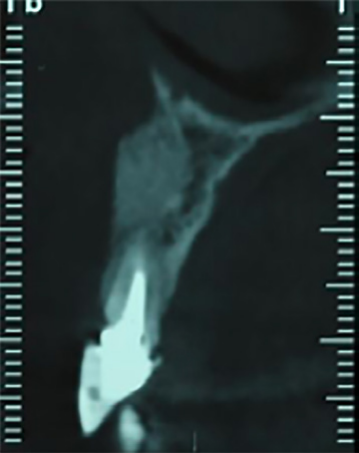
The CBCT view of the grafted site after 8 months.

**Figure 5:**
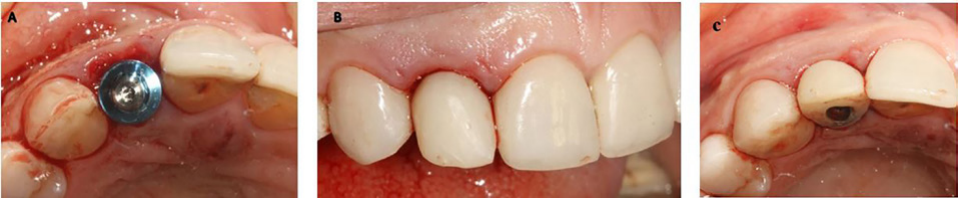
(A) Placement of an Osseotite implant in the extraction socket. Immediate provisional composite-based crown from the frontal (B) and occlusal views (C)

After eight months, the grafted site healed without any complications. A CBCT view showed a favorable reconstruction of the grafted area (Figure 4). Local anesthesia with 2% lidocaine (1:100,000 epinephrine) was delivered. Without flap elevation, the lateral incisor was extracted atraumatically using a periotome. The extraction socket was thoroughly curetted for complete debridement of the granulation tissue and then irrigated with normal saline solution. Osteotomy site preparation was performed, and a self-threaded titanium implant (3.75×11.5 mm, Osseotite®, Biomet 3i, Florida, United States) was inserted at the extraction socket. Insertion torque value was 40 N/cm2, and favorable primary stability was achieved from the residual alveolar bone. The implant platform was placed 2 mm apical to the cementoenamel junction of the right maxillary central incisor (Figure 5A). The gap between the implant surface and the labial wall of the socket was filled with xenograft particles (cerabone®, Botiss Biomaterials GmbH, Zossen, Germany).

A temporary cylindrical abutment was tightened on the implant, and a composite-based provisional crown (Dentocrown, Itena, Villepinte, France) was fabricated on the temporary cylinder to make a screw-retained provisional restoration (Figure 5B). The occlusal assessment was made, and any centric and eccentric contacts were eliminated. Amoxicillin, 500 mg (q8h for seven days), gelofen, 400 mg (q6h for seven days), and 0.2% CHX mouthwash (twice daily for a week) were prescribed. The patient was placed on a soft diet for two months.

**Figure 6:**
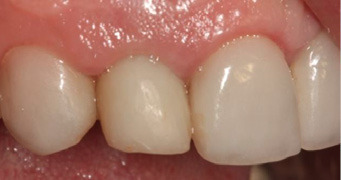
Clinical view 4 months after implant placement.

**Figure 7:**
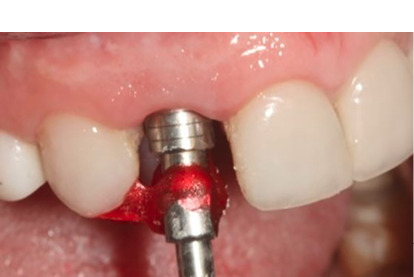
An open-tray impression coping was used for the final impression.

**Figure 8:**
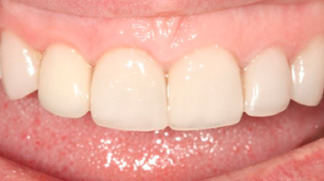
A definitive screw-retained metal ceramic restoration was fabricated 4 months after implant placement.

A regular follow-up was performed every month. After four months, the site healed without any complications (Figure 6), and the periapical radiograph showed no bone loss and radiolucency around the implant. The provisional restoration was removed, and an open tray impression was taken using the putty/wash technique (Imprint^TM^, 3M, Minnesota, United States) (Figure 7). The implant abutment torqued to 25 N/cm2, and a definitive screw-retained metal-ceramic restoration was delivered (Figure 8).

**Figure 9:**
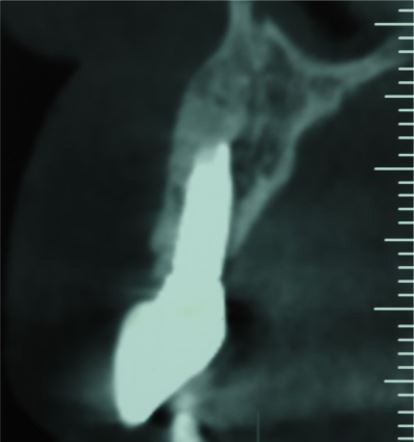
The radiographic view 5 months after implant placement.

Regular follow-ups were performed every three months during the first year and then every six months

The five-year follow-up examination revealed stable and healthy peri-implant soft tissue, and the patient was satisfied with the esthetic outcome.

## Discussion

Several studies have demonstrated that guided bone regeneration is successful for horizontal augmentation.^6^ This paper reported the case of a hopeless maxillary lateral incisor and the concave osseous architecture of the buccal bone plate. For the bone regeneration procedure, a special flap was designed to preserve the interdental papilla and free gingival architecture, which reduced the risk of the papilla and gingival recession in the future. Implant replacement following tooth extraction and immediate provisionalization led to improvements in the appearance and psychological status.

Different studies have indicated that immediate implant placement is a successful and predictable treatment.^7-10^ Implants placed in the extraction socket exhibited high survival and success rates comparable to those for implants placed in healed sites.^11^ Immediate implant placement and provisionalization provide optimal esthetic by preserving the hard and soft tissue architecture.^12,13^ However, placing an implant in the fresh extraction socket cannot prevent physiologic modeling/remodeling that occurs in the socket wall. Width and height changes of the buccal plate are more pronounced than those in the lingual plate.^14-16^

In a study by Chen et al,^17^ s fractured maxillary central incisor was extracted, and an implant was placed immediately in combination with provisionalization and simultaneous GBR. The 18-month follow-up showed a high implant success rate and satisfactory esthetic outcomes.

In another study by Levin et al,^18^ simultaneous with immediate implant placement, GBR was performed, and a screw-retained provisional restoration was delivered. At one-year follow-up, all the 29 implants were successfully osseointegrated with excellent stability and minimum bone loss.

According to a recent review, the recession of the facial mucosal margin was more frequent for immediate implant placement.^19^ A case-series study with a one-year follow-up demonstrated that immediate implant placement with a flapless procedure was associated with significantly less mid-facial recession than the flap surgery.^20^ Different studies have suggested that flapless implant insertion reduces soft tissue inflammation and marginal bone loss and enhances the peri-implant tissue vascularization.^21-23^

Different studies are available on the management of the marginal gap between the implant surface and the fresh extraction socket walls during immediate implantation. For horizontal marginal defect ≤2 mm, there is no need to use regenerative procedures.^24^ Since studies have demonstrated that deproteinized bovine bone has an osteoconductive effect on new bone formation^25^ and reduces bone resorption in post-extraction human sockets,^26^ Cerabone was used for the treatment of the marginal gap after implant placement in the patient presented.

In the esthetic zone, provisional restorations have several benefits. Assessment of esthetic, phonetic, and occlusal functions before the fabrication of final implant restorations and establishment of a natural and esthetic peri-implant mucosal tissues and interdental papilla are some of the advantages of provisional restorations.^27^

## Conclusion

This clinical report presented the esthetic and functional reconstruction of a fractured maxillary right lateral incisor using minimally guided bone regeneration, immediate implant placement, and provisionalization. Maximum enhancement and preservation of hard and soft tissue architecture, lack of anterior edentulous period, and optimum improvement of the appearance and confidence are the advantages of this treatment modality. However, careful patient evaluation and selection are essential for a successful outcome.

## Authors’ contributions

AF: surgeon, FP: scientific writer, SY: supervisor of team. All authors have read and approved the final manuscript.

## Competing interests

The authors declare that they have no competing interests (financial and non-financial).

## Ethics approval

Our study is a case report and before study initiation, informed consent was obtained from the patient. All the procedures that has been done for the patient are evidence based.

## Authors’ information

Abdulreza Fattahian: Master clinician in implant dentistry, Private practitioner, Tehran, Iran. E-mail: afattahian@yahoo.com

Farzaneh Poursafar: Periodontology resident, Tehran University of Medical Sciences, Tehran, Iran. E-mail: farzaneh.poursafar70@gmail.com

Siamak Yaghobee: Associate professor, periodontology Department, Tehran University of Medical Sciences, Tehran, Iran.
